# Short-term efficacy and safety of transcleral lutein iontophoresis in patients with intermediate amd and reticular pseudodrusen: A retrospective study

**DOI:** 10.1038/s41433-026-04731-7

**Published:** 2026-07-24

**Authors:** Mutasem Elfalah, Enrico Borelli, Paola Marolo, Michele Reibaldi, Vincenco De Angelis, Carlo De Rosa, Francesco Iatì, Niccolò Castellino, Sandrine Anne Zweifel, Yacoub A. Yousef, Daniela Bacherini, Andrea Lucisano, Adriano Carnevali, Robert Rejdak, Giuseppe Giannaccare, Vincenzo Scorcia, Mario Damiano Toro

**Affiliations:** 1 Department of Special Surgery, Ophthalmology Unit, The University of Jordan, 11941 Amman, Jordan; 2 Department of Surgical Sciences, Section of Ophthalmology, University of Turin, 10126 Turin, Italy; 3 UOC di Oftalmologia, Ospedale A. Cardarelli, 80131 Naples, Italy; 4 Department of Ophthalmology, University of Naples Federico II, 80131 Naples, Italy; 5 Department of Ophthalmology, University of Catania, 95123 Catania, Italy; 6 Department of Ophthalmology, University Hospital Zurich, 8091 Zurich, Switzerland; 7 University of Zurich, 8006, Zürich, Switzerland; 8 Department of Surgery/Ophthalmology, King Hussein Cancer Centre (KHCC), 11941 Amman, Jordan; 9 Eye Clinic, Neuromuscular and Sense Organs Department, Careggi University Hospital, 50134 Florence, Italy; 10 Department of Medical and Surgical Sciences, Azienda Ospedaliero-Universitaria R. Dulbecco, University Magna Græcia of Catanzaro, Catanzaro, 88100 Italy; 11 Department of General and Pediatric Ophthalmology, Medical University of Lublin, 20079 Lublin, Poland; 12 Eye Clinic, Department of Surgical Sciences, University of Cagliari, 09124 Cagliari, Italy

**Keywords:** Macular degeneration, Outcomes research

## Abstract

**Background:**

There is limited data available on the efficacy and safety of Transscleral Lutein Iontophoresis (TSLI) in patients with intermediate age-related macular degeneration (iAMD) and reticular pseudodrusen (RPD).

**Methods:**

This retrospective multicentre study evaluated the functional and morphological outcomes of TSLI in patients with stage 2 and stage 3 iAMD with RPD. Autofluorescence (AF), Optical Coherence Tomography (OCT), Best Corrected Visual Acuity (BCVA), Intraocular Pressure (IOP), Macular Sensitivity (MS) and adverse events, were recorded at baseline, 1 month and 3 months follow-up after treatment.

**Results:**

Thirty eyes from 30 patients, (15 ♂: 15 ♀), mean age (70.3 ± 7.8 years) were included. BCVA improved significantly, from mean baseline 65.6 ± 8.6 ETDRS letters to 66.9 ± 8.0 at 1 month and 69.1 ± 7.8 at 3 months (ANOVA, *p* < 0.0001, respectively), along with MS, (ANOVA *p* < 0.001). There was a significant increase of stage 1 RPD (*p* = 0.002) and a decrease in stage 3 RPD (*p* = 0.020). The Outer Nuclear Layer (ONL) thickness increased significantly (ANOVA, *p* < 0.0001). No adverse events were recorded.

**Conclusion:**

TSLI appears to be an effective, safe technique in patients with iAMD and RPD, leading to structural and functional improvements in short-term follow-up.

## Introduction

Age-related macular degeneration (AMD) is a progressive chronic disease and a leading cause of vision loss and legal blindness in western countries [1, 2]. It is estimated to affect about 288 million people by 2040, in people over 55 years of age. Classification of AMD distinguishes between early, intermediate and advanced AMD as by the Age-Related Eye Disease Study (AREDS) [3, 4]. While early and intermediate stages are by definition non-neovascular “dry”, its late stages are classified into two clinical phenotypes: dry AMD (d-AMD), also known as atrophic or non-exudative AMD and wet AMD (nAMD), neovascular or exudative [5]. AMD is a multifactorial disease, with several risk factors: age, smoking, diet, genetic and environmental factors, as well as oxidative stress. Although the main aetiology of AMD is not completely understood, oxidative stress appears to be a main driver of AMD progression, with the retinal pigment epithelium (RPE) and its associated micro-environment as the primary site of pathology. RPE cells are a non-dividing system, extremely prone to oxidative stress in relation to their enormous metabolic activity (exposure to intense irradiation, hyperbaric oxygen), which develop structural and functional changes linked to aging [6, 7].

In the last decades, the introduction in the clinical practice of anti-vascular endothelial growth factor (VEGF) injections has changed the natural history of the nAMD, dramatically reducing the visual loss due to the macular neovascularisation (MNV) [3]. Conversely, no specific treatment is available to avoid the progression of dAMD into its advanced stage [8, 9].

Indeed, d-AMD, which accounts for 80-90% of all cases of AMD, is characterised by a progressive atrophy of the outer layers of the retina (retinal pigment epithelium and overlying photoreceptors), leading to a late-stage disease, defined as geographic atrophy (GA), which progresses to severe central vision loss [8, 9]. As reported by the Age-Related Eye Disease Study (AREDS) and AREDS2 study, dietary supplementation and general lifestyle modification could reduce the risk of AMD progression in high-risk patients affected by dAMD [10, 11]. Recently, for late-stage d-AMD a complement protein C3 inhibitor, approved by the US Food and Drug Administration (FDA), known as Pegcetacoplan, has shown to slow the growth of GA lesions both in phases 2 and 3 clinical trials, with an acceptable safety profile but with no benefit on visual acuity [12].

However, no specific therapies that slow the progression from earlier and intermediate stages of the disease into the late stage of dAMD are available. For these reasons, several groups have focused their research on this field, trying to propose new strategies for the treatment of this diffuse and devastating disease. Among other characteristics, reticular pseudodrusen (RPD) are findings present in the early and intermediate stages of dAMD, accompanied by RPE changes and drusen [13]. Indeed, it was demonstrated RPD contributes to faster progression of iAMD late AMD [14–16]. For these reasons, a treatment aimed at the regression of RPD could be mandatory in order to prevent the progression to late AMD.

Lutein and zeaxanthin are important carotenoids, macular pigments (MPs), which are highly concentrated in the macula, compared to the peripheral retina; they play a key role in maintaining macular anatomy and function. Compared to healthy individuals, AMD patients have significantly lower levels of MP pigment [17]. Several studies, including the CAREDS, the Blue Mountain Eye Study, and the AREDS2, have reported that higher dietary lutein and zeaxanthin intake reduces the risk of AMD progression. Its stringent supplementation increases the level of MP in patients with AMD [18, 19]. Accordingly, AREDS 2 study demonstrated the efficacy of adding Lutein 2 mg and its structural isomer zeaxanthin to antioxidant supplementation in reducing the rate of progression from intermediate to late AMD [20]. Moreover, Age-Related Macular Degeneration Preferred Practice Pattern 2019 concludes that antioxidant vitamin and mineral supplementation as per the Age-Related Eye Disease Study (AREDS2) should be considered in patients with intermediate or advanced AMD.

Specifically, lutein in the macula filters blue light, quenches free radicals and supports vision. As a result, it may play a role in prevention of age-related eye diseases. Oral supplementation of these carotenoids has been thoroughly studied. As demonstrated by Feng et al., data reported in literature are not completely superimposable due to the treatment regimen, the compliance of patients and bioavailability through the gastrointestinal tract. Therefore, researchers started to evaluate different ways of administration. In 2017, Lombardo et al. studied a novel method for in situ delivery of lutein via the sclera to the human retina, using a iontophoresis device, demonstrating effective transscleral delivery of Lutein to retina in a ex vivo model [21]. To date, despite CE-approval of the method and its spreading in clinical practise, only very limited data about its efficacy and safety are available.

For this reason, the aim of this study was to evaluate functional and anatomical outcomes and the rate of adverse events in patients with early and intermediate stage AMD who underwent Lutein Iontophoresis scleral administration in the short term.

## Materials and methods

This is a retrospective, single-arm  multicentre study, which included patients aged between 55 and 80 years-old with the diagnosis of early to intermediate stage dAMD (based on AREDS criteria) and presence of RPD (based on classification by Zweifel et al., Querques et al.) who underwent Lutein Iontophoresis scleral administration [3, 4, 15, 22], from September 2023 to August 2024. The study comprises four ophthalmological departments (Ospedale Cardarelli, Naples, Italy; University of Turin, Turin, Italy; University of Catania, Catania, Italy; University Magna Grecia of Catanzaro, Catanzaro, Italy). All patients had a minimal follow up duration of 3 months. The study was conducted in compliance with the tenets of the Declaration of Helsinki, approved by Regione Calabria Ethics Committee (protocol number 273/2024) and registered at ClinicalTrials.gov (Identifier: NCT07420361). All study participants provided written informed consent prior to the procedure and for the use of their clinical data for scientific research in an anonymised manner.

We excluded patients with: evidence of GA or MNV in the included eye, any prior or any ongoing medical or surgical treatment of the included eye (including intravitreal injections, oral supplementation of lutein, zeaxanthin and or high dietary intake of antioxidants), as well as smokers (consummation of more than 20 cigarettes per day), patients with a history of treatment with drugs with a well-known possible ophthalmic side-effect, known eye diseases such glaucoma, acute or chronic uveitis, advanced cataract, stage 4 dry eye disease or any opacities of the ocular media that do not permit high-quality imaging examinations, manifest refraction outside of -6 and +4 dioptres, and BCVA < 40 EDTRS letters. In cases where both eyes were eligible, the eye with the worse BCVA at baseline was selected as the study eye. If both eyes have the same BCVA, it was recommended to select the right eye as the study eye. After obtaining informed consent and after the screening visit, at the Baseline (Day 0) all patients were evaluated with a complete ophthalmic examination. BCVA was assessed using glasses with refractory values obtained using an autorefractometer (NT-530/510®, NIDEK Inc., San Jose, CA, USA), using Early Treatment Diabetic Retinopathy Study (ETDRS) charts [23]. Intraocular pressure measurements were acquired using the same device. Additionally slit lamp examination, fundus examination (by indirect ophthalmoscopy) were assessed in all patients. As imaging protocol, all patients were evaluated using Spectral Domain Optical Coherence Tomography (SD-OCT, Revo Optol, OPTOPOL Technology Sp. z.o.o., Zawiercie, Poland), fundus autofluorescence (FAF). FAF images were performed using Spectralis HRA + OCT (Heidelberg Engineering, Heidelberg, Germany). Microperimetry was performed using MP-1 (Nidek Technologies, Padova, Italy). All patients were evaluated at 1-month and 3-month follow-up, with a complete ophthalmic examination, including SD-OCT, FAF, and microperimetry.

### TS-Lutein Iontophoresis application technique

At baseline, each patient received one application of scleral iontophoresis. The following steps were performed:

1. After applying a drop of anaesthetics (0.4% oxybuprocaine, Novesina, Novartis, US) onto the eye to treat, a passive electrode (return electrode) was placed onto the patient’s forehead after cleaning the area with 70% alcohol.
2. The passive electrode and the active electrodes (including the ocular applicator) were then connected to the power supply battery (K-IONO, Offhealth SpA, Italy), via a cable;
3. The ocular applicatior was placed on the open eye to treat and filled with 0.1% lutein ophthalmic solution (Lipo + , Offhealth SpA, Italy).
4. The current intensity was set for 2.5 mA for 4 min.

At the end of the procedure, the ocular applicator was removed and the eye was rinsed with balanced salt solution to remove any excess Lutein. The patient was then asked to remain supine on the operating bed for 5 min.

### Imaging (Fundus autofluorescence, spectral domain-OCT)

FAF imaging was used at baseline and during the follow up visits to identify the possible appearance of GA (defined as a hypo-autofluorescence area). In order to investigate the outer retinal morphology, including the assessment of RPD stages, spectral domain-OCT (SD-OCT) imaging was performed at baseline and during the follow-up as established by Querques et al. [22]. The thickness of the outer nuclear layer (ONL) was measured at baseline and during the follow-up visits. RPD were classified as previously described by Zweifel et al. [15] and Querques et al. [22] RPD were graded as stage 1 if diffuse deposits of hyperreflective material between the RPE and the inner/outer segments (IS/OS) boundary was assessable. Grade 2 was defined by presence of deposits/material that altered the IS/OS boundary. Deposited material that appeared thicker and more conical in appearance piercing through the IS/OS boundary was graded as stage 3 RPD. Reduction of the deposits supposedly by absorption and/or migration within the inner retinal layers was categorised as stage 4 [22], aligning the classification used in this study with recently published papers on novel methods applied in patients with AMD, such as subthreshold laser, or photobiomodulation [24–26]. The ONL thickness associated to each RPD was assessed manually, with the mean value of thickness measurements included in the statistical analysis. The grading of the RPD and the retinal assessments were executed by two independent and experienced readers. When the graders did not reach a consensus, the senior author made the final decision.

The central macular thickness (CMT) was assessed using the Optopol Revo NX (Optopol, Poland). For optimal choroidal visualisation, enhanced depth imaging (EDI) structural OCT was performed. The choroidal thickness (ChT) was evaluated subfoveally by manually measuring the distance between Bruch’s membrane and the sclerochoroidal thereby delineating the inner and outer boundaries of the choroid. For follow up, the scans were aligned with the previous ones, utilising the follow-up function of the Optopol Software (version: 11.3).

### Microperimetry

Microperimetry was performed to evaluate the central retina’s sensibility. To evaluate retinal sensibility, all participants received an introduction training to microperimetry. The pupils were dilated and patients underwent dark adaptation in advance of the scotopic microperimetry. As part of the training the patients performed a fast test for learning. To confirm the anatomical position of the fovea the combined central infrared reflectance image and the horizontal B-scan SD-OCT scans were uploaded to the MP-1S software. The retinal vessel arcades and the optic nerve head were used for orientation in registration of the fundus real-time image of the MP-1S. Microperimetry-stimuli were positioned around the fovea (Goldmann size V, 200 ms, 4-2 strategy, background luminance 0.0032 cd/m2 with the grid centred on the anatomical position of the fovea). During each timepoint the mean overall retinal sensitivity of the macular area (MS) was measured, as well as the fixation percentage in the central 2 and 4 degrees.

### Clinical outcome measures

Primary and secondary outcomes are reported as below:

structural parameters:

- Outer retinal morphology using OCT and FAF assessing: RPD type, ONL thickness, ChT, CMT and changes in FAF

functional parameters:

- Mean BCVA (ETDRS) over the course of the follow-up
- Mean macular sensitivity (MS) over the course of the follow-up.

safety parameters:

- Monitoring of adverse events over the course of the follow up.

### Statistical analysis

For statistical analysis, functional parameters such as BCVA, MS and fixation percentage in the central 2 and 4 degrees, and structural parameters such as RPD type, CMT, ONL thickness, ChT, and FAF changes and IOP were assessed at baseline and at 1 month and at 3 months follow-up, after scleral iontophoresis. The data was evaluated and presented as means ± standard deviations (SD). The mixed model repeated measures analysis of variance (ANOVA) with η p 2 as an effect size indicator was used to determine whether there were any significant differences between the baseline, 1 and 3 months of the follow-up. For post hoc comparison, Bonferroni test was used. A *p*-value less than 0.01 was considered statistically significant (including correction for multiple comparisons). Statistical analysis of the data was performed using GraphPad Prism version 10.3 (GraphPad Software, San Diego, CA, USA).

## Results

Thirty eyes of 30 patients (mean age 70.3 ± 7.8years, median 71.5, range 55–80 years) fulfilled the criteria of our study and were included. All patients were Caucasians, 15 females and 15 males. The fellow eye of 4 patients was affected by nAMD and received anti-vascular endothelial growth factor (VEGF) intravitreal injection. Twelve eyes were affected by early AMD (AREDS stage 2), 18 eyes were classified as intermediate AMD (AREDS stage 3). The fellow eye of 14 patients was affected by dAMD. Demographics and clinical findings of our study cohort are reported in Table 1.

**Table 1:** Demographics and clinical findings of the study group at baseline.

	patients (*n* = 30)
**sex (male : female)**	15:15
**age (years) [+/- SD]**	70.3 + /- 7.8
**laterality OD:OS**	13:17
**AREDS stage 2:3**	12:18
**mean number of RPD Type 1 (** + **/- SD)**	0.53 + /- 0.50
**mean number of RPD Type 2 (** + **/- SD)**	6.76 + /- 1.61
**mean number of RPD Type 3 (** + **/- SD)**	2.26 + /- 1.01
**mean number of RPD Type 4 (** + **/- SD)**	0.33 + /- 0.47

*n* number, *SD* standard deviation, *AREDS* Age Related Eye Disease Study, *RPD* Reticular Pseudodrusen.

At the baseline, BCVA ranged from 52 and 80 ETDRS (mean 65.6 ± 8.6 EDTRS letters). BCVA showed mild mean improvement at 1-month compared to baseline values (a mean gain of 1.3 ETDRS letters), (65.6 ± 8.6 EDTRS letters and 66.9 ± 8.04 at baseline and 1 month follow-up, respectively) (ANOVA, p < 0.0001). Between baseline and 3-month follow-up a statistically significant improvement (65.6 ± 8.58 EDTRS letters and 69.7 ± 7.8 at baseline and 3 months follow-up, respectively) (ANOVA, p < 0.0001) was observed.

In detail, we observed a mean improvement of 1.3 ± 1.4 ETDRS letters and 4.1 ± 2.3 ETDRS letters at 1 and 3 months follow-up, respectively. Compared to baseline, at 3 months follow-up more than half of the patients (56%) gained from 1–5 letters, while 30% gained from 6–10 ETDRS letters. No patient gained more than 10 ETDRS letters during the follow-up. Four patients (13%) showed no changes in BCVA during the follow-up. While at 1 month postoperatively 3 patients (10%) lost 1–5 ETDRS letters, at final follow up no patient showed any loss in terms of ETDRS letters compared to baseline. Over the follow-up period of 3 months an increasing proportion of patients had a higher gain in BCVA. These results are summarised in Table 2.

**Table 2:** Descriptive analysis of visual function (BCVA) at baseline, 1 month and 3 months after TSL-Iontophoresis.

	Mean change of ETDRS letters ± SD	ETDRS letters gained 1 to 5, *n* patients (%)	ETDRS letters gained 6-10, *n* patients (%)	ETDRS letters no change, *n* patients (%)	ETDRS letters lost 1 to 5, *n* patients (%)	ETDRS letters lost 6 to 10, *n* patients (%)	ETDRS letters change ± of 11 or more, *n* patients (%)
**Baseline vs. 1 month**	1.3 ± 1.4	25 (83)	0 (0)	0 (0)	5 (16)	0 (0)	0 (0)
**1 month vs. 3 months follow-up**	2.8 ± 2.0	23 (76)	3 (10)	1 (3)	3 (10)	0 (0)	0 (0)
**Baseline vs. 3 months follow-up**	4.1 ± 2.3	17 (56)	9 (30)	4 (13)	0 (0)	0 (0)	0 (0)

*BCVA* Best Corrected Visual Acuity, *TSL* *Iontophoresis* Transcleral Lutein Iontophoresis, *ETDRS* Early Treatment of Diabetic Retinopathy Study, *SD* Standard Deviation, *%* percentage.

IOP did not show significant changes during the follow-up. At baseline, the ONL thickness was 50.13 ± 4.66 microns (µm). The ONL-thickness showed statistical significance difference at 1-month compared to baseline values (50.13 ± 4.66 and 54.1 ± 4.39 at baseline and 1 month follow-up, respectively) (ANOVA, *p* < 0.0001), while a statistically significant difference was observed between baseline and 3-month follow-up (50.13 ± 4.66 and 60.1 ± 3.65 at baseline and 3 months follow-up, respectively) (ANOVA, p < 0.0001).

Furthermore, ChT showed changes over time, yet on a barely significant level at baseline vs. 3 months (214.57 ± 17.53 µm vs. 216.83 ± 17.16, *p* = 0.0069), and between 1 and 3 months follow up (215.67 ± 17.08 µm vs. 216.83 ± 17.16, *p* = 0.047) (Table 2.). MS showed significant differences between the baseline and 1-month follow-up (12.6 ± 1.97 dB vs. 13.26 ± 1.94 dB, *p* = <0.0001) and between the baseline and 3-month follow-up (12.6 ± 1.96 dB vs. 17.55 ± 2.37 dB, *p* < 0.0001) and 1 months and 3 months (13.26 ± 1.94 dB vs. 17.55 ± 2.37 dB, *p* < 0.0001), (Table 2.). Mean fixation percentage calculated within the central 2° (centred on the fovea) was 38.11 ± 6.09. 2% at the baseline and did not significantly change during the follow-up (*p* = NS); also mean fixation percentage calculated within the central 4° (centred on the fovea) did not show significant changes during the follow-up (*p* = NS) (Table 2). All the data related to the anatomical and functional variables between baseline and 1-month and 3 –month follow-up after the treatment are summarised in Table 3.

**Table 3:** Comparison of anatomical and functional variables between baseline, 1-month and 3 months follow-up after treatment.

	Baseline	1 month follow-up	3 months follow-up	ANOVA	baseline vs. 1 month	baseline vs. 3 months	1 month vs. baseline
	*Mean ± SD*	*Mean ± SD*	*Mean ± SD*	*p* value*	*p* value§	*p* value§	*p* value#
**BCVA (LogMAR)**	65.6 ± 8.6	66.9 ± 8.04	69.77 ± 7.82	<0.0001	<0.0001	<0.0001	<0.0001
**CMT (µm)**	228.13 ± 17.5	229.27 ± 11.45	228.23 ± 11.11	0.12	>0.9999	>0.82	0.001
**ONL thickness (µm)**	50.13 ± 4.66	54.1 ± 4.39	60.1 ± 3.65	<0.0001	<0.0001	<0.0001	<0.0001
**ChT (µm)**	214.56 ± 17.53	215.67 ± 17.08	216.83 ± 17.15	0.0014	0.09	0.006	0.047
**MS (dB)**	12.6 ± 1.96	13.26 ± 1.93	17.55 ± 2.37	<0.0001	<0.0001	<0.0001	<0.0001
**Fixation percentage (2°)**	38.11 ± 6.09	38.33 ± 5.87	37.73 ± 5.67	0.2	0.6	>0.9999	0.32
**Fixation percentage (4°)**	80.63 ± 6.83	81.3 ± 6.55	81.63 ± 6.68	0.09	0.03	0.25	>0.9999

*SD* standard deviation, *BCVA* best-corrected visual acuity, *IOP* intraocular pressure, *CMT* central macular thickness, *ChT* choroidal thickness, *ONL* outer nuclear layer, *MS* retinal sensitivity. *Analysis of Variance (ANOVA) for paired samples. §Comparison with baseline using ANOVA for paired samples with Bonferroni post-hoc analysis. #Comparison with 1-month follow-up using ANOVA for paired samples with Bonferroni post-hoc analysis.

**Fig. 1:**
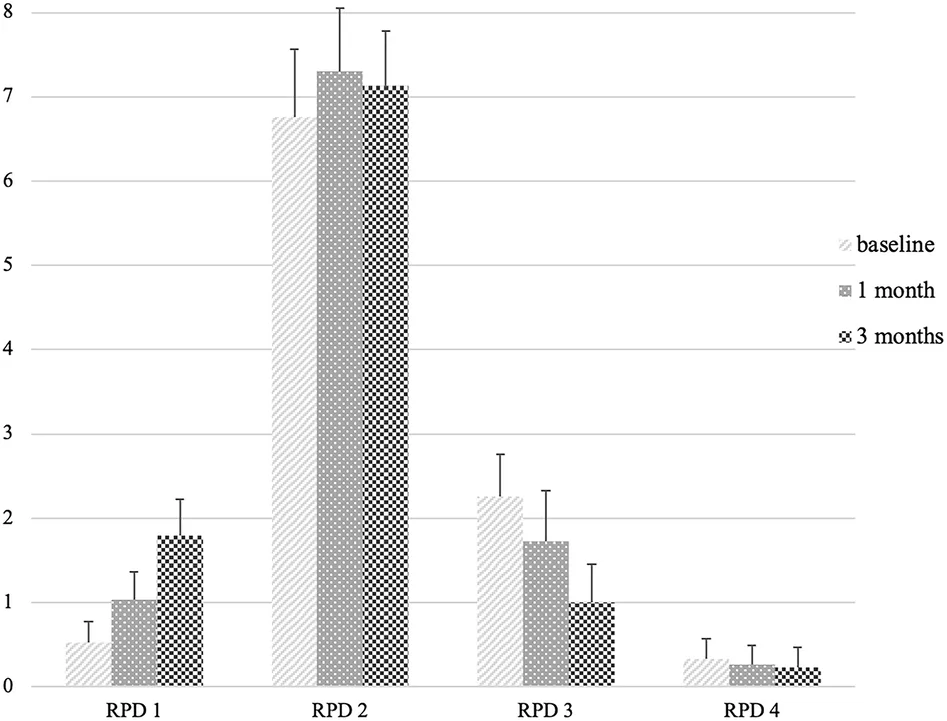
Distribution of RPD stages after trans-scleral-Lutein-Iontophoresis, x-Axis signifies the mean number of reticular pseudodrusen (RPD) at baseline and at 1- and 3-months follow up. The y-axis represents the stages of RPD 1-4. The associated indicator to each bar represents the respective standard deviation. RPD 1 (first collum) increased numerically over the course of follow up, while RPD 3 (third collum) decreased over time. The mean numbers of RPD 2 and RPD 4 (second and fourth collum) remained stable over time.

Using structural OCT, we identified a mean of 9.9 ± 2.2 RPD per patient. The distribution of RPD among the RPD stages changed after the treatment (p < 0.001). At the baseline, 0.53 ± 0.50 RPD were classified as Stage 1, 6.78 ± 1.61 as Stage 2, 2.27 ± 1.02 as Stage 3, and 0.33 ± 0.48 as Stage 4. At 1-month follow-up, 1.03 ± 0.67 RPD were classified as Stage 1, 7.30 ± 1.49 as Stage 2, 1.73 ± 1.20 as Stage 3, and 0.27 ± 0.45 as Stage 4. At 3-month follow-up, 1.80 ± 0.85 RPD were classified as Stage 1, 7.13 ± 1.28 as Stage 2, 1.00 ± 0.91 as Stage 3, and 0.23 ± 0.43 as Stage 4 (Fig. 1).

This accounted for a significant increase of Stage 1 RPD during the follow-up (*p* = 0.002), no significant changes of Stage 2 RPD (*p* = 0.909), but a significant decrease of Stage 3 RPD (*p* = 0.020) and no significant changes of Stage 4 RPD (*p* = 0.630). In detail, there was an increase of 52% of stage 1 RPD (16 vs. 31 of stage 1 RPD of all patients at baseline and 3 months follow-up, respectively). In stage 2 RPD there was an slight increase of 5% of number of drusen of all patients (203 vs. 214 at baseline and 3 months follow-up, respectively). 56% of stage 3 RPD (38 out of 68 stage 3 RPD of all patients) showed a reduction during the 3-month follow-up. For stage 4 RPD a slight reduction of 30% in number of total drusen was observed between baseline and 3-months follow-up, respectively (10 vs. 7), Fig. 1.

No adverse events were monitored over the 3 months follow-up period. Apart from dAMD, stage 2 and 3 no further retinal pathologies were detected in multimodal imaging during the follow-up period. Specifically, neither macular neovascularisation (MNV), nor atrophic lesions were identified in treated eye. A large majority of patients reported redness of the eyes (*n *= 27, 77%) at day 1 post TSL, treatment, however this diminished over time. Ocular discomfort (i.e. itching and foreign body sensation), or pain was reported by a small to moderate number of patients (*n* = 5, 16%) had ocular discomfort at day (1). Two patients, (7%) reported pain at day (1). Both findings resolved in the majority of cases during the follow-up period. At day 1 of follow-up 2 patients (6%) reported photophobia and 4 patients (13%) declared symptoms of lacrimation which resolved by their next follow up visit. (Table 4).

**Table 4:** Patient reported side effects after treatment.

	at day 1	at month 1	at month 3
**pain, n patients (%)**	2 (7)	0 (0)	0 (0)
**redness, n patients (%)**	23 (77)	3 (10)	2 (6)
**discomfort, n patients (%)**	5 (16)	1 (3)	1 (3)
**photophobia, n patients (%)**	2 (6)	0 (0)	0 (0)
**lacrimation, n patients (%)**	4 (13)	0 (0)	0 (0)

The number and percentage of patients (n), (%) who experienced either pain, redness or discomfort at day 1, month 1 and months 3 after surgery was recorded. Redness of the eye was a common reported finding in the early time period after the TSL-application. An effect that diminished over the observed follow-up periode.

## Discussion

In this retrospective multi-centre study, we evaluated the efficacy and short-term safety of scleral lutein iontophoresis in patients diagnosed with stage 2 to 3 AMD. In our cohort we observed an increase in functional parameters such as BCVA and MS, as well as anatomical changes such as increase of ONL, reduction of stage 3 RPD and increase of stage 1 RPD. No lasting ocular or systemic adverse events were reported upon completion of the follow up period.

Vision loss caused by advanced stages of AMD has a profound impact on an individual’s quality of life and contributes to wider socioeconomic issues. The costs of productivity loss and of rehabilitation for blindness constitute a significant economic burden for an individual, their family, and society globally [7–10]. Thereby, effective strategies of secondary prevention and development of novel diagnostics and treatments for halting AMD progression or ideally improving stage 2 and 3 AMD would be highly desirable. The levels of macular pigments such as Lutein are significantly lower in patients with AMD, when compared to healthy controls [17]. One main function of the macular pigments, i.e. Lutein and Zeaxanthin, is to protect the outer retina against photo-induced damage by short-wavelength light. The macular pigment’s antioxidative properties ameliorate the high amount of free oxidative radicals in this highly active metabolic tissue by induction of antioxidant enzymes, such as superoxide dismutase, improving the rate of macular pigment optical density [27–29]. Apart from its anti-inflammatory properties, Lutein is hypothesised to strengthen the blood retina barrier by inducing tight junction repair in photo-stressed retinal pigment epithelium [30]. The choroidal vasculature, on the other end of the outer blood retina barrier and its interaction with Bruch’s membrane and the retinal pigment epithelium are highly relevant in the pathogenesis of AMD. Yet, apart from the multimodal imaging data of the retrospective pilot study by Lombardo et al. [21], and this retrospective study, there is currently no prospective clinical or histological evidence that elucidate on how transscleral lutein iontophoresis influences this highly relevant interface. Future studies incorporating OCTA imaging and ideally histological work-up could shed light on this important topic.

Patients with intermediate AMD can benefit from changes in lifestyle including daily supplementation of Lutein and Zeaxanthin, as a means of secondary prevention, reducing the risk of progression to advanced AMD by 20% as demonstrated in the AREDS 2 trial [10]. More precisely, dietary supplementation of lutein, 10-20 mg/day may confer greater protection against photo damage in patients at risk of progression of AMD [31]. Yet, the effect of oral supplementation is limited by several factors, such as patient compliance, the necessity for continuous supplementation (lifelong) and the limited absorption of this carotenoid through the digestive route [27, 31, 32]. Indeed, the plasma concentration of lutein after supplementation varies considerably among individuals and may be influenced by type of supplementation, duration of intake, amount of fat in the diet, concomitant ingestion of fibre, age, genetic factors and concomitant therapies. Thereby, dietary supplementation of Lutein may not be the most optimal route of administration. For this reason, new strategies are being investigated, which aim to enrich macular pigment faster than current conventional methods and improve patient compliance.

TSL-Iontophoresis is non-invasive, fast drug delivery technique with a low learning-curve, with recent application in ophthalmology. It allows for transport of charged molecules in fluid formulation to a target tissue by applying an electric current [33, 34]. By establishing an electrical gradient between a passive and an active diode, charged molecules (i.e. Lutein) can be transported transclerally to the retina. Ex-vivo studies demonstrated the efficacy of TSL-Iontophoresis, based on Resonance Raman Spectroscopy, depicting an increase of Lutein in Henle fibre layer and outer photoreceptor segments in donor eyes treated with TLS-Iontophoresis compared to controls [21]. Data of in-vivo application of TSL-Iontophoresis is limited. To date, there is one published pilot study on the efficacy and safety of the procedure available. In that prospective pilot study with 3 months follow-up, Lombardo et al. demonstrated an improvement of BCVA after treatment in a small cohort of patients with dAMD [2], without assessing the structural changes after treatment. While Lombardo et al. found a statistically significant improvement of BCVA at one week after the procedure, with slight reduction over the course of follow-up, we noted a persisting significant improvement of BCVA at 1 month and 3 months follow-up. The BCVA in EDTRS letters increased from 65.6 ± 8.6 letters at baseline to 66.9 ± 8.04 at 1 month and to 69.7 ± 7.8 at three months follow-up. We observed a mean improvement of 1.3 ± 1.4 ETDRS letters and 4.1 ± 2.3 ETDRS letters at 1 and 3 months follow-up, respectively. These results are in comparable with Lombardo et al. who reported an increase of 3 ± 10.0 ETDRS letters at 3 months follow-up in their pilot study [2]. Upon closer inspection of our cohort, 86% of patients had an increase in BCVA at 3 months follow-up (56% of patients gained from 1-5 letters, while 30% gained from 6–10 ETDRS letters). Four patients (13%) showed no changes in BCVA during the follow-up. At final follow up no patient showed any loss in terms of ETDRS letters compared to baseline. No direct comparison can be performed between both study populations. Lombardo et al.’s study included patients with advanced AMD (30%), contributing to a smaller (*n* = 9) more heterogenous study group than the data of the patients of our study presented here, including 30 patients with only stage 2 and stage 3 AMD.

Apart from dietary supplements and TSL-Iontophoresis, other approaches to treat intermediate AMD are being investigated such as subthreshold laser treatment (SLT) as recently reported by Querques et al. In their study all patients were treated in an extrafoveal area of 1.27 mm2 using end-point management SLT and followed for 3 months [24]. Best-corrected visual acuity was 0.140 ± 0.09 LogMAR at the baseline and no changes were observed during the follow-up (*p* = 0.232). In our series we observed an improvement of BCVA at 1 and 3 months follow-up. A possible explanation for the lack of functional improvement after SLT in Querques et al.’s study lies in the fact that the treatment was performed extrafoveally, while in TSL-Iontophoresis the entire posterior pole is treated, as shown by the ex vivo study by Lombardo et al., where scleral iontophoresis was effective in enriching the macula of donor eyes with lutein forty minutes after treatment. As outlined by Querques et al., taking into account their functional and structural findings, further studies with SLT including treatment of the fovea, larger area with longer follow-up are warranted to assess SLT’s role as an intervention in intermediate AMD. The LIGHTSITE III trial which investigated the outcome of Photobiomodulation (PBM) in patients with early to intermediate AMD showed statistically significant improvements in BCVA at 4 months follow-up, on a similar level to our findings at 3 months follow-up [26]. In detail, the mean BCVA was 70.7 ± 0.55 letters (LIGHTSITE III trial) and 65.6 ± 8.6 (TSL-Iontophoresis) at baseline and 74.8 letters at 4 months (LIGHTSITE III Trial) and 69.77 ± 7.8 and 3 months (TSL-Iontophoresis) follow-up, respectively. The fact that the LIGHTSITE III trial had a four months follow-up visit but no three months visit reduces comparability of both studies. Yet, within the limitations of the small study cohort of this TSL-Iontophoresis study, *n* = 30 eyes of *n* = 30 patients vs. *n* = 148 eyes, *n* = 100 patients (LIGHTSITE III trial), the results indicate a similar functional result after one single treatment of TSL-Iontophoresis (while PBM requires repetitive treatments) during the observed short-term follow-up of three months.

In concordance with the findings of Querques et al. on the outcome of SLT in intermediate AMD, we found a significant increase of ONL thickness from 50.13 ± 4.66 μm and 59.30 ± 13.50 μm in the treated area at baseline to 60.10 ± 3.65 μm and 67.75 ± 15.52 μm at 3 months follow-up, respectively [24]. Similarly, we found concordance in the changes of RPD stages after treatment. After treatment, we observed a significant increase in the number of stage 1 RPDs, and a significant decrease of stage 3 RPDs from baseline 0.53 ± 0.50 to 1.80 ± 0.84 and from 2.26 ± 1.01 to 1.00 ± 0.90 at three months follow-up respectively compared to 0.35 ± 0.59 to 2.30 ± 2.18 and 3.30 ± 4.35 to 1.15 ± 2.56 as reported by Querques et al. Regression of RPD can be observed in advancing AMD followed by outer retinal atrophy (i.e. thinning of the ONL thickness), as reported by Spaide et al. [24, 35]. Curiously, we found a suspected remodelling of RPD stages after TSL-Iontophoreses, with an increase of stage 1 RPD, and a decrease of the later stage 3 RPD, accompanied by an increase in ONL thickness, as previously reported for SLT by Querques et al. [24]. While the discussed extrafoveal SLT treatment did not yield a significant increase in visual acuity, the structural findings were comparable to those reported here after TSL-Iontophoresis. As reported in previous studies this is hypothesised to represent a sign of regression of the disease, indicating a reduced risk of developing advanced forms of AMD after treatment. The LEAD study, demonstrated subthreshold nano second laser (SNL) to be safe in carefully selected patients but to not significantly reduce the rate of progression of intermediate AMD to late AMD. Furthermore, patients with RPD treated with SNL were found to have a worse outcome, than patients without RPD. However, no detailed morphological and functional analysis of treated RPD was performed in the LEAD study. In contrast to the findings of the LEAD study Querques et al.’s and our findings indicate a structural improvement after the respective treatment. This might be due to the different mechanism of action of the different methods SLT and TSL Iontophoresis vs. SNL on the RPE cells. In addition to the apparent structural improvements, we observed functional improvements in the domains of BCVA and MS within the short term follow up period of this study. Determining whether the primary aims of this treatment, which include secondary prevention, reducing risk of progression to advanced AMD or even regression of AMD can be achieved and maintained, requires prospective long-term studies with the inclusion of larger patient cohorts, as well as accompanying histological correlation.

Despite the encouraging results our study has several limitations such as the retrospective design, the low number of patients included in our cohort and the short-term follow-up. However, this is the first retrospective study reporting on the efficacy and safety of TSL-Iontophoresis in patients with stage 2 and stage 3 dAMD with RPD, demonstrating promising results and thereby indicating a potential benefit for this novel approach.

Another limitation is the absence of volumetric measurements of volume of RPD. While in contemporary research the measurement of drusen volume is of high relevance, this initial study on short term efficacy and safety was based on the readily established classification and outcome parameters utilised in similar studies. Yet, further parameters including drusen volume should be incorporated in future studies on TSL-Iontophoresis to provide quantitative data further detailing the treatment response of the individual RPD subtypes, based on the expanding literature of RPD classification.

TSL-Iontophoresis appears to be effective in inducing RPD regression with respect to RPD stage and ONL thickening, as well as conferring benefits in functional parameters such as BCVA and MS, whilst maintaining an adequate safety profile over the observed short-term period. Nevertheless, further prospective randomised clinical trials with longer follow-up duration, different regimen of re-treatment and larger sample size are required to demonstrate reproducibility, long-term effects on the morphological and functional outcomes, and the safety in patients with intermediate AMD and RPD treated with TSL-Iontophoresis. Whilst the provision of TSL-Iontophoresis as secondary prevention or even as a potential treatment is yet to be determined, our findings indicate that it may have a role in future clinical practice.

## Summary

### What was known before

- intermediate AMD appears to be receptive to modulation by non invasive methods

### What this study adds

- trans scleral lutein iontophoresis appears to positively affect patients with intermediate AMD in a small study group with short follow-up

## Data Availability

The data that support the findings of this study are not openly available due to reasons of sensitivity and are available from the corresponding author upon reasonable request and located in controlled access data storage.
